# Lean Healthcare Tools for Processes Evaluation: An Integrative Review

**DOI:** 10.3390/ijerph18147389

**Published:** 2021-07-10

**Authors:** Letícia Bianchini de Barros, Letícia de Camargo Bassi, Laura Passos Caldas, Alice Sarantopoulos, Eliete Boaventura Bargas Zeferino, Vinicius Minatogawa, Renata Cristina Gasparino

**Affiliations:** 1 School of Nursing, University of Campinas, Campinas 13083-887, Brazil; lehbassi6@gmail.com (L.d.C.B.); la.passos@outlook.com (L.P.C.); grenata@unicamp.br (R.C.G.); 2 Faculdade de Ciências Médicas, Universidade Estadual de Campinas, Campinas 13083-887, Brazil; alice_sarantopoulos@hotmail.com; 3 Hospital de Clínicas, University of Campinas, Campinas 13083-888, Brazil; elietebb@hc.unicamp.br; 4 Escuela de Ingeniería en Construcción, Pontificia Universidad Católica de Valparaíso, Valparaíso 2340000, Chile; vinicius.minatogawa@pucv.cl

**Keywords:** quality improvement, process assessment, health care, health services, workflow, total quality management

## Abstract

Several health services have used lean healthcare to seek continuous improvement of their processes. Therefore, it is important to investigate the evidence available in the literature about the most used lean tools in the health area to review processes and the main results achieved by the researchers. As an integrative literature review methodology was used, it was conducted in five databases, using the descriptor “quality improvement” and the keyword “Lean Healthcare”. A total of 33 complete articles were selected for analysis. The most recurrent tools were: define, measure, analyze, improve and control (DMAIC); value stream map (VSM); suppliers, inputs, process, outputs, customers analysis (SIPOC), Ishikawa Diagram and 5S. Through the analysis of waste, different interventions were implemented and the main results achieved were reduction in times (processing, waiting, cycle and total), costs, workload and increase in the number of calls. The findings enabled the identification of the main lean tools used in the health area to achieve better results. In particular, we highlight recent studies that have explored the lean six sigma healthcare approach. The results, in addition to contributing to the literature, will also assist managers in choosing the best tool to achieve continuous improvement in hospitals and other health services.

## 1. Introduction

Quality care in health services guarantees an increase in the quality of life for the population, resulting in greater socioeconomic development [1]. The difficulty in maintaining effective and efficient processes constitutes an important barrier to the prevention of diseases and to minimize the suffering of those who are already ill. Therefore, dealing with this problem involves governance and public policy actions that ensure the patient’s entry to the health service, as well as the continuity of their treatment by this service [2].

In order to resolve procedural problems, we have a health management philosophy called lean healthcare (LH), which enables the removal of rework, waste and unnecessary procedures. The LH is a healthcare adaptation of the lean philosophy created by Eiji Toyoda, founder of Toyota Motor Company, a Japanese car company [3].

These tools have been applied in several processes in the healthcare area, helping performance and facing challenges such as staff shortages, rising costs and providing high quality services, considering current financial constraints [4]. Lean six sigma, as well as lean healthcare, has also been applied in healthcare services and has shown to be very promising due to its character of addressing operational problems in complex environments [4].

With the use of this philosophy, many services report the great potential for operational performance improvement [5]. However, to assimilate and embed tools that lead organizations to a lean operation is not a trivial task. In fact, many companies fail in this process [6,7]. The implementation of lean tools goes beyond its philosophy, requiring methodology.

This scenario becomes even more complex when the adaptation is not just for a new company, but for a new sector. A clear example of this complexity is lean healthcare. Adapting to health context requires caring for patient’s life, before addressing productivity concepts. The literature offers a wide range of Lean tools applications in health services, considering necessary idiosyncrasies [8].

Articles on the use of lean tools have increased significantly in recent years [9]. At this point, the lean healthcare literature presents, however, an important gap, that is, which tools are being applied health services and which are results of such applications. Nevertheless, lean tools framework is quite broad [10]. Thus, analyzing all tools within all possible processes would make results too broad, not enabling us to elucidate the results. Instead, we aim to understand which lean healthcare tools are currently being used for process reviewing and find the results of the implementation of such tools pointed out in the literature.

Process review tools provide current situation diagnosis in hospitals and other healthcare facilities [11]. Hence, addressing the proposed aim may reveal important results for both scholars and practitioners. Our work responds to an important gap in the literature; specifically, it connects tools and their results. For academics and healthcare professionals, such a contribution offers a basis for developing lean healthcare tools practical applications. Moreover, we found methodological limitations in several studies, requiring attention for further studies.

Important organizational (decrease in the length of stay and lead time), social (improvement of patient centered services and improved communication between staff, patients and family), clinical (infection rate reduction and shorter treatment time) and economic (increase in revenue and cost reduction) impacts are revealed in the application of lean tools in the healthcare system. We argue that these impacts are interconnected, reinforcing the idea that lean methodology assistance achieves better results that go far beyond specific changes in work processes [12].

To fill the presented gap, this article presents the methodological framework and the guiding question of the study. In this step, it is explained how the search in the databases was conducted. From there, the included articles are presented and, soon after, the results are discussed based on the literature. Finally, the theoretical contributions and practical implications of this research are presented, as well as the limitations of this study.

## 2. Materials and Methods

As a methodological framework, an integrative review was used, which condenses the literature about a particular phenomenon of interest in order to provide a synthesis of knowledge from selected studies and analyzed in a systematic way [13,14].

For this review, six steps were followed: (1) establishment of the research question, (2) sampling or searching the literature, (3) categorization of studies, (4) evaluation of studies that were included in the integrative review, (5) interpretation of results and (6) presentation of the review [13].

▪ Step one: Establishment of the research question

In step one, to elaborate the research question, the “Population, Intervention, Comparison and Outcome”—PICO [15] strategy was used and the following question was elaborated: Which tools of the lean healthcare philosophy are being used most for the review of work processes and what are the results that are being achieved, in the health area?

▪ Step two: Sampling or searching the literature.

For the conduction of the second stage, the identification, selection, eligibility and inclusion of the articles was carried out following the Preferred Reporting Items for Systematic review and Meta-Analysis Protocols—PRISMA [16] recommendation. The search was carried out according to the following inclusion criteria: complete articles, published in English, Portuguese or Spanish, that had application of lean tools in healthcare services and that has been published between 2015 and 2019. This period was chosen in order to obtain a recent analysis at the current scenario of lean applications. The following exclusion criteria were considered: theses, dissertations, books, reviews, opinion articles and editorials; these are characterized as gray literature and articles that did not apply the tools but only indirectly cite them in their analyses.

The search was carried out in five databases, which are: Virtual Health Library (VHL), National Library of Medicine National Institutes of Health (Pubmed), Scopus, Cumulative Index to Nursing and Allied Health Literature (CINAHL) and Embase.

Due to the absence of a specific controlled descriptor for the theme lean healthcare, prior to defining the combination used in the present study between the descriptor and the keyword, the authors assessed which descriptors were being used most in scientific publications. Based on this and with the help of a librarian, search strategies were being developed and tested and, after the options were exhausted, the one that resulted in the largest number of articles was chosen.

The search strategy included the descriptors “Quality Management” and “Process Assessment (Health Care)”, as well as their synonyms in Portuguese and Spanish: “Gestión de la Calidad”, “Gestão da Qualidade”, “Evaluación de Proceso (Atención de Salud)” and “Avaliação de Processos (Cuidados de Saúde)”. All of them associated with the keyword “Lean Healthcare” in the title or in the summary. The descriptors were used with the Boolean operator “AND” between them.

In the search strategy formulated for the VHL, descriptors based on the DeCS terms were used. In Pubmed and Scopus, MeSH terms were applied, as well as their synonyms. At Embase and CINAHL, their peculiarities were respected and the terms EMTREE and CINAHL titles were used, respectively.

**Figure 1:**
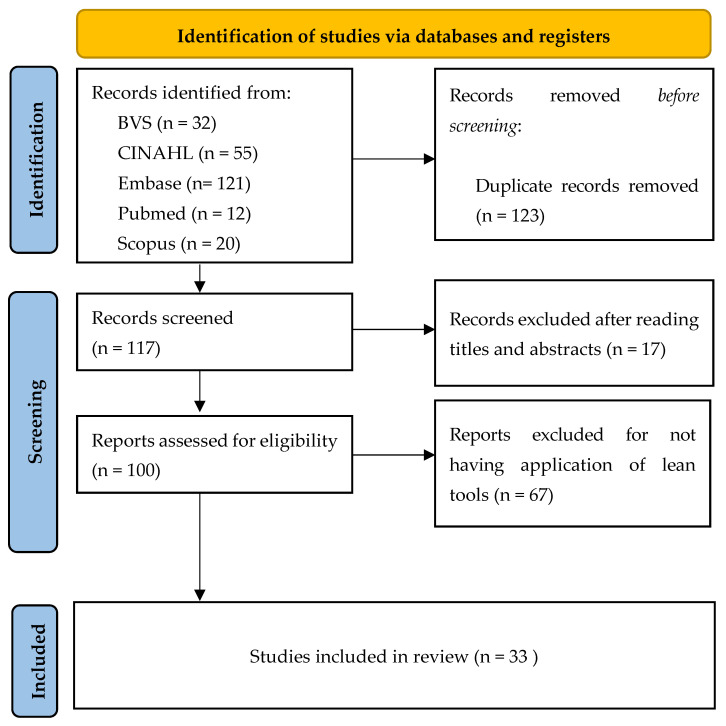
Flowchart of the data collection process adapted from the PRISMA recommendations [18].

The search was carried out by two researchers, independently and the results were exported to the reference manager EndNote^®^ (Figure 1).

▪ Step three: Categorization of studies

In step three, to manage the included studies and decide which information would be selected, a validated “Data Collection Instrument” was used, which includes the following variables: base/portal, title, journal, authors’ names, country research, language, year of publication, institution hosting the study, study design, objective, selection and sample size, inclusion/exclusion, data treatment, interventions performed, tools used, study duration, results and level of evidence [17].

▪ Steps four and five: Evaluation of studies that were included in the integrative review.

In the fourth step, a critical analysis of the studies included in the review was carried out and in step five, the main results found in the research were interpreted. In the last stage, in addition to the conclusion, they were identified as a reference in the integrative review [13,15,16,17]. The ethical aspects of this study were preserved, as all authors of the analyzed articles were adequately cited.

## 3. Results

The search strategy carried out by this integrative review provided some important results. We condensed the literature review into 33 articles. All the analyzed articles present recent applications of lean tools in healthcare and the main results achieved by the researchers. All the articles included answered the inclusion criteria described in the methodological section. Among the articles, 67 were excluded during the researchers’ evaluation because, although these articles mentioned the use of lean tools, they did not describe their practical applications, an important criterion to fulfill the study objective.

**Figure 2:**
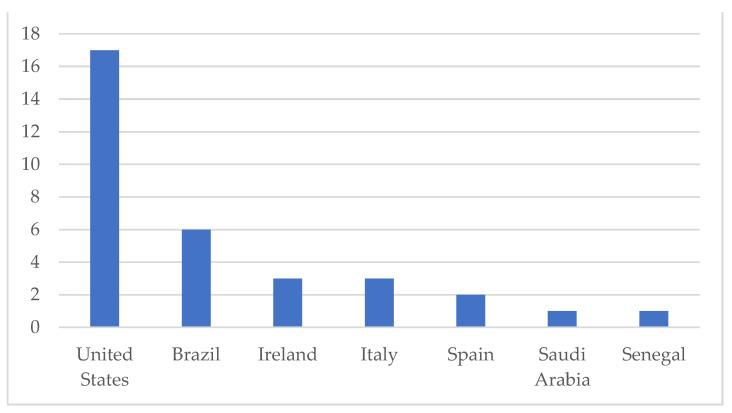
Countries where the articles were produced.

We explored many different respected search databases, which provided us high quality papers. Hence, papers analyzed are also indexed in Journal of Citation Reports (considering Web of Science database) or ScimagoJR (considering Scopus database). Regarding the papers, eight of them (24.3%) explore lean six sigma applications, while 25 (75.7%) lean healthcare applications. As for the language, 32 (96.9%) were written in English and only 1 (3%) in Spanish [19]. Most surveys (17, 51.5%) were conducted in the United States. The other countries that published on the topic were shown in the Figure 2.

**Figure 3:**
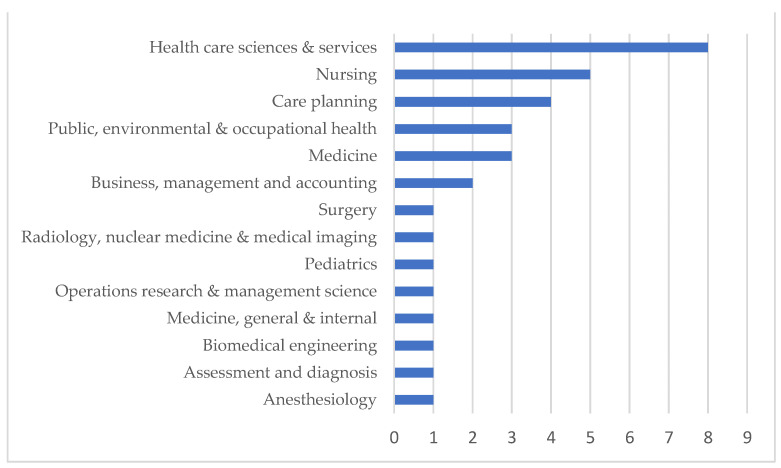
Academic fields.

Another significant result regards the publications’ academic fields. We classified such data according to the Web of Science and ScimagoJR records. Two fields condense most of the journals in which the analyzed papers were published. The health care sciences and services field presents eight articles (24.3%, while the nursing field presents five (15.2%) analyzed articles. The complete data can be seen in Figure 3. Regarding the journals, the articles were published in 26 different journals, all described in Appendix A. 

**Figure 4:**
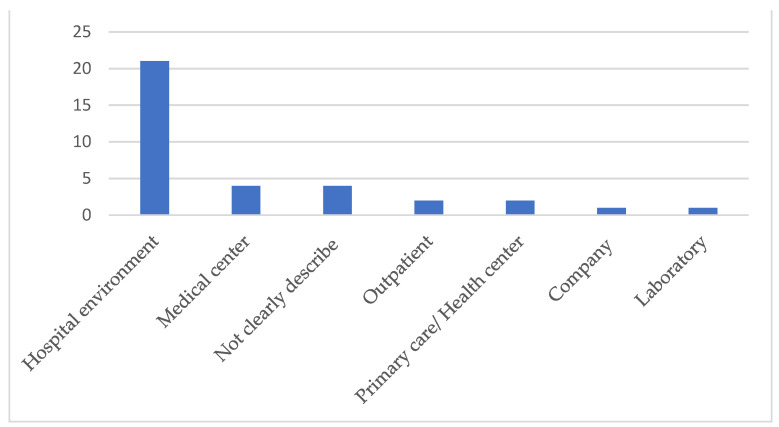
Locations where tools were applied.

Of the 33 analyzed studies, 21 (63.3%) were carried out in the hospital environment, four (12.1%) did not clearly describe the place of study [20,21,22,23] and in two (6.1%) articles, the study was conducted in more than one place [24,25]. The other places where the studies were carried out are presented Figure 4.

**Figure 5:**
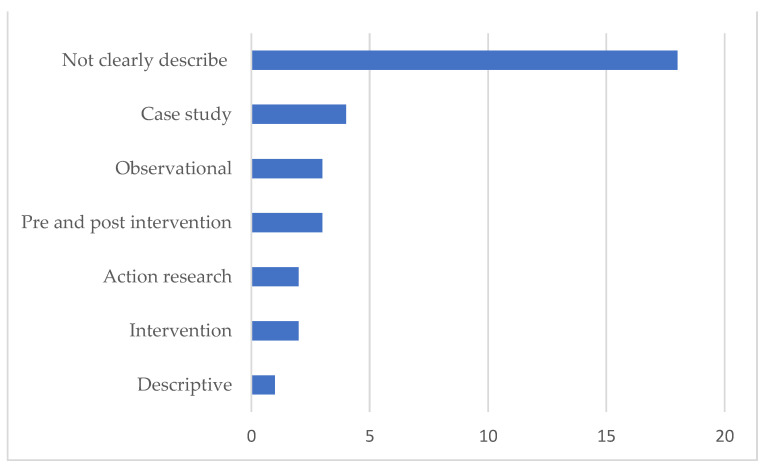
Study designs adopted by researchers.

It is noteworthy that in nine (27.3%) of the articles, the calculation used to determine the sample size was found, while approximately nine (27.2%) of the surveys did not contain a description of the sample selection method. As for the study design, 18 (54.5%) publications did not clearly describe which one was used and the most used design was case study (4, 12.1%). The other designs used in the research were shown in Figure 5.

**Figure 6:**
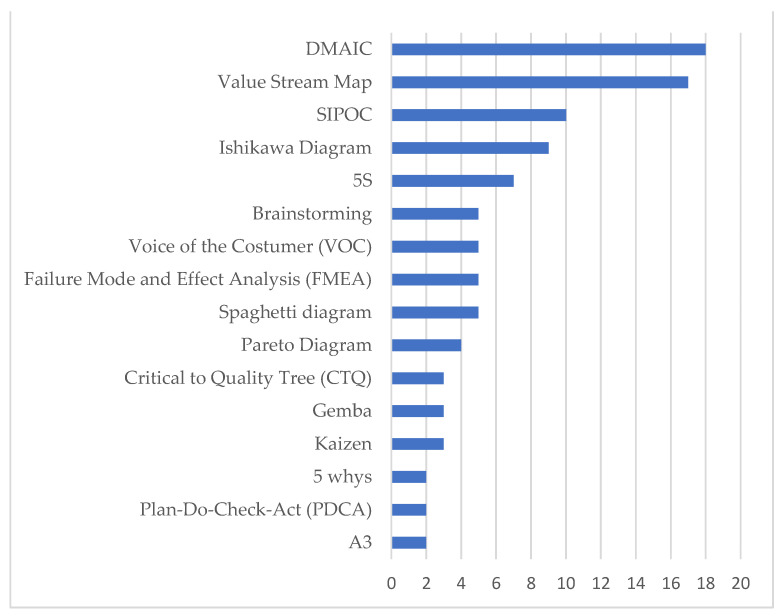
Main tools used by the articles included.

In the analysis of the studies, 30 different tools used by the authors were found. Approximately 50% of them were applied only once by different articles, as shown in the chart below (Figure 6). The define, measure, analyze, improve, and control (DMAIC); value stream map (VSM); suppliers, inputs, process, outputs, customers analysis (SIPOC); Ishikawa diagram and 5S tools were the most used in the diagnosis of waste and intervention applications and the possible, implement, challenge and kill (PICK); responsible, accountable, consulted and informed (RACI); specific, measurable, achievable, realistic, time frame (SMART); analytic hierarchy process (AHP); Poka Yoke; flowchart; Heijunka; matrix exchange tool; Kanban; Gant diagram; strengths, weakness, opportunities, threats (SWOT); First in first out (FIFO); just in time and map of steps were described only once.

**Figure 7:**
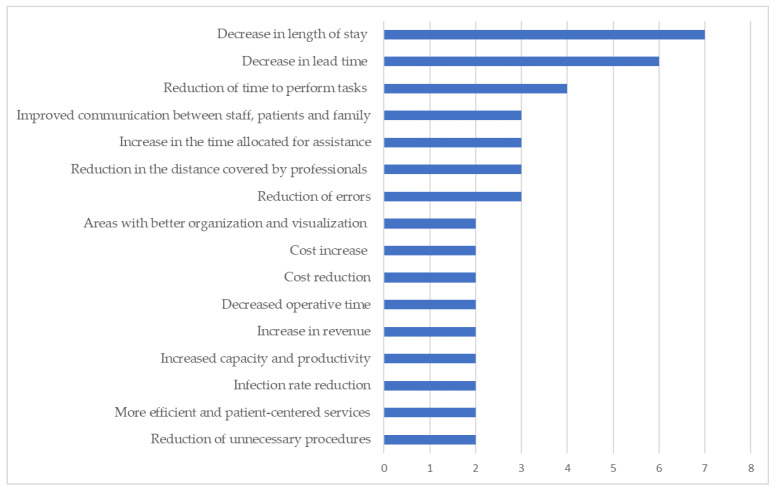
Graph of the main results obtained from the included studies.

With the application of the tools, different results were achieved. Those that appear the most are related to the length of stay and lead times of patients in the units studied. All results are listed in Appendix A. In Figure 7, the most frequent results obtained by the studies in total can be seen.

Appendix A shows the other variables collected from the publications included in the review: title, journal, year, objective, their respective tools, interventions, results and level of evidence.

## 4. Discussion

According to the literature, lean healthcare’s main target is to improve management and organization of health services. According to lean production principles, company must define what is value based on customer’s point of view, reduce waste by implementing a continuous flow, in order to mitigate inventories, delays and interruptions. It is also recommended to adopt pull production, that is, produce only when customer requires it. Institutions must also strive for perfection through continuous improvement [8,26].

Still following lean principles, it is important to analyze the value chain. Activities that add value are those that make the product or service more valuable to customers. Necessary activities do not add direct value to customers; however, institutions must maintain and improve them. Lastly, activities which do not add any value must be eliminated [27].

These principles and waste are the theoretical foundations that constitute lean manufacturing philosophy [28]. With this, it is already known that lean is a philosophy that has been increasingly implemented in the health area over the past years [9]. With the results obtained through this review, it was possible to verify which tools are being used more in this area and which are the main results achieved.

The studies found were published, predominantly, in the English language, probably due to the fact that the majority were carried out in the United States, one of the pioneers in the application of lean healthcare [9]. The second country that published the most research on the subject was Brazil, demonstrating that lean has been the target of growing interest in the country. These Brazilian authors have also published in international journals, which shows that the results achieved in Brazil have supported research around the world [21,29,30,31].

The interest by Brazilian researchers may have connection with their public health system. Such a system has as mission to provide free and in large scale healthcare services. Thus, there is a need to investing in improvements related to: patient satisfaction, responsiveness, reliability and security of the services provided. Lean healthcare application can help institutions achieve these goals [1].

In the health area, lean has been used predominantly in the hospital environment; however, it has expanded and contributed to the achievement of improvements in other levels of services, demonstrating that the philosophy is useful for the most different organizations [32,33].

This could have an interesting impact on future studies. Lean healthcare can help the healthcare area, as it did the industry, to understand its processes as a supply chain, which is in line with the concept of healthcare networks. [34].

The fields of study revealed that 15% of the articles analyzed were published in nursing journals. This may be related to the fundamental role of nurses in health services management and the interest in lean thinking, which causes a paradigm shift related to the healthcare sciences and services.

In the qualitative analysis of the articles found, there were difficulties in classifying the study design, since most did not describe this item clearly. In addition, some authors used more than one design to characterize this variable [29,35,36]. This is an important gap found in recent studies in the area of lean healthcare; a design suggestion for research involving this topic would be evaluation research, conceptualized as process analysis aimed at obtaining useful information about a program or process, to support decision-making related to it [35].

It is important that researchers follow a rigorous methodological process that allows replication of the study and obtaining similar results [36], especially when these results are positive. However, when the study design and other method variables are not clearly defined, they end up contributing to the publication of articles without a high level of evidence.

Another gap found is related to the sample, both in terms of calculation and the selection of participants. Scholars claim that the calculation and method of selecting the sample must be carried out clearly, containing the inclusion and exclusion criteria, thus facilitating the replicability of the research by other authors [37].

From the selected articles, it was possible to observe that the vast majority aimed to identify waste and implement solutions in search of improvements within the units. From this, it was possible to observe which were the most used tools in the health area: DMAIC (18 studies), VSM (17 studies), SIPOC (10 studies), Ishikawa diagram (9 studies) and 5S (7 studies).

DMAIC was used in 54.5% of the studies and through its five phases (define, measure, analyze, improve and control), this tool makes it possible to define the goals of a project, as well as to identify the problem and its causes. DMAIC, as well as the Toyota Motor Company’s A3 Report, can help establish a method for achieving improvement [38]. These results demonstrate a greater frequency in combining lean methodology along with six sigma tools for processes reviewing in healthcare. The concept of lean healthcare has been evolving in the literature towards a lean six sigma healthcare approach.

Followed by DMAIC, VSM was chosen by 48.4% of the authors and this tool helps to map the flow of processes and identify activities that do not add value to the client. From this, it is possible to eliminate waste and increase the efficiency of the process [39].

Activities that do not add any value are also called as waste. Classifying waste in lean methodology comprises the following topics: (1) overproduction, which results in excess of products in stock; (2) over processing, a process that is too sophisticated to the detriment of simpler and more efficient approaches; (3) inventory: excess of stored products, which results in high cost; (4) movement: excessive movement of people; (5) transport: excess transport of goods or information, which causes an increase in time, effort and cost; (6) waiting: very long periods of inactivity of goods, information or people; (7) defects: errors in the information process, product quality problems or poor delivery performance and (8) waste of human talent: people are seen only as operators and not as process specialists [40].

The SIPOC matrix was present in 30.3% of the surveys and in 27.2% of them it was used in conjunction with DMAIC. SIPOC is a tool that helps in understanding the suppliers, inputs, outputs and customers of a given process [41].

The Ishikawa diagram, which also had an important participation in publications, was used in 24.2% and had a solitary participation in only one article [42]. Generally, the Ishikawa diagram is used in conjunction with other tools, as it aims to identify and analyze the causes of a problem [43].

With respect to 5S, used in 21.2% of studies, this tool focuses on the organization of institutions and aims to improve the work environment, reduce time that does not add value, increase productivity, improve quality and maintain a clean and organized environment for new changes [44]. Other numerous tools were used by the authors of the mapped studies and, from them, waste and its possible causes were identified, enabling the implementation of interventions that contributed to the improvement of results.

Among these interventions, we can highlight reorganization of the physical structure [19,30,39,41,45], review of work processes [38,46,47,48,49], development and standardization of documents [29,50,51,52,53] and team training [24,25,41,54,55], among others. A single article that aimed to evaluate the current process of a service did not present any type of intervention [22].

From the implementation of these strategies, several positive results were achieved and, with the use of DMAIC, the ones that stood out the most were decreased lead time, reduced length of stay and decreased costs [21,30,38,48,55].

The combination of DMAIC and SIPOC resulted in an increase in the number of patients seen, less waste related to costs, reduced turnover time, length of stay and cycle time. This combination usually occurs because DMAIC requires the use of other tools to be applied, so the combination with SIPOC helps one better understand the workflow in the initial phase [32,33,51,56,57].

VSM, used in 17 articles, helped in the diagnosis of waste of the studied units. After the application of these tools, interventions were carried out by the authors, obtaining in several cases a reduction in the workload, length of stay, treatment, waiting and, consequently, a reduction in lead time. In contrast, a study found that after implementing changes, there was an increase in costs [47].

Studies have shown that the use of other tools in conjunction with VSM has made it possible to expand the benefits of mapping [58]. It was observed that the 5S tool was cited in 18.2% of the articles that also used the VSM and results such as increased time for patient care, restructuring of the physical structure, visual management, reduction of lead time and improvement financial performance were also achieved [21,22,41,56,59]. 

Three studies [39,47,59] are noteworthy as they present a reduction in the distance traveled by professionals as a result of their interventions. These three used the VSM tool and two of them combined them with the spaghetti diagram; from this, we can infer that the combination of these two tools contributes to a better use of the physical space of the health units.

The outcomes that stood out the most in the evidence were related to time, be it processing, waiting, cycle, permanence or total. Most studies showed improvement in these times, after the implementation of interventions based on the causes of waste previously mapped [21,23,25,29,31,32,38,42,46,47,48,49,50,54,56,57,60].

In contrast, despite knowing that improving “times” can contribute to increasing patient satisfaction, this topic has clearly not been addressed in the studies. The Lean Institute Brazil emphasizes that, in addition to reducing times, it is extremely important to keep in mind that perfect care must not only be agile, but also fair, efficient, effective, safe and always centered on the patient [61].

In this regard, three articles showed improved communication between professionals, patients and family members and two studies reported more patient-centered care after using lean tools. It is emphasized that, in order to achieve continuous improvement, in addition to the application of tools, it is necessary to continuously search for new knowledge, skills and behaviors and that, for this purpose, the establishment of a lean culture throughout the institution is essential. In addition, it is necessary for managers and leaders to become facilitators and mentors, involving the whole team in identifying and solving problems based on an attitude of continuous improvement [27].

Reviewing different lean healthcare tools and their applications, we observed different impacts. There are organizational and economic impacts regarding institutions. In addition, we also found social and clinical impacts affecting work teams and patients. Moreover, the interconnection between such impacts is also noteworthy. From an organizational point of view, tools help to change healthcare service flows and, as an effect of this, for example, one can obtain a “decrease in length of stay and lead time”, “increased productivity”, “reduction of errors” and time “increased in the time allocated for assistance” [20,21,32,50,51,56]. 

Changes at the organizational level, following lean thinking, also require a cultural adaptation [62]. Organizational and cultural changes result in direct impacts on clinical aspect of patients. For this matter, we can highlight, for instance, effects such as “infections rate reduction” and “shorter treatment time” [31,55,63]..

These clinical effects naturally benefit the patient. However, from a social point of view, there are other important impacts: “improved communication between staff, families and patients” and “patient-centered services”. At the end of this chain of impacts, economic benefits can also be achieved. In this sense, there are effects of “increase in revenue” and “costs reduction” [21,38,48]. 

Finally, following lean philosophy, it is possible to understand that the activities are part of a value chain and that the improvements arising from the implementation of this philosophy go far beyond specific changes in work processes [12]. Our review strengthens the perception that the effects of lean healthcare occur in an interconnected chain and that waste reductions provide improvements that impact professionals, patients and families, as well as institutions.

## 5. Conclusions

According to the evidence found in the literature, the lean tools that have been most used in the health area are DMAIC, VSM, SIPOC, Ishikawa Diagram and 5S. Combining these tools, the researchers obtained positive results, such as time reduction (processing, waiting, cycle, permanence and total), cost reduction, improvement in the workload and increase in the number of consultations.

From this, it is concluded that the use of these tools has helped in the improvement of the processes in the health services. However, although it is known that lean philosophy offers methods for application, for the analysis of its results, it is relevant to emphasize the importance of researchers adopting a well-designed study and sample design for scientific proof and the replicability of their applications.

### 5.1. Study Limitations

There is no controlled descriptor for the “lean healthcare” themes and for the tools used within this philosophy. Therefore, the search strategy had to be developed from the descriptors that appear most in scientific publications. Based on this, the search strategies were developed and tested and, after the options were exhausted, the one that resulted in the largest number of articles was chosen. The absence of specific descriptors for the topic in question culminates in a lack of standardization in publications, which may have impaired data recovery.

The intention of the authors of the present study was to classify the level of evidence of all articles; however, given the absence of a clear description of the drawings used in the studies, this classification was only partially performed.

### 5.2. Contributions to the Area

This research offers recommendations and contributions to the health area, especially regarding the possibility of including controlled descriptors specific to the topic and carrying out future research. There is a need to develop specific descriptors for this topic, which has been widely used, so that there is greater standardization by researchers in the use of these terms and, consequently, easier retrieval of articles.

In addition, it is recommended that the authors adopt, in their manuscripts, a clearer description of the study design and a suggestion would be evaluation research with process analysis.

The description of how the sample calculation and the sample selection method were carried out also needs to be improved. In addition, conducting well-designed clinical trials is necessary to increase the level of evidence of studies on the subject.

A second impact of this study was the grouping of the main lean tools and their respective results, making it possible to clearly visualize, in a single article, the results obtained through the combination of these tools, by the most recent studies on the subject. Thus, this review can be extremely useful for researchers and managers who are starting on this journey.

It is important to emphasize that the conduction of research in health services outside the hospital environment is still very incipient and has the potential to generate improvements. In addition, and perhaps most importantly, from the moment that lean philosophy was born, it had the main intention that the processes must generate value for the client; therefore, incorporating the patients’ perceptions of the care received is extremely important.

### 5.3. Future Studies

The positive results achieved by these studies show that lean healthcare is effective for improving processes in healthcare services. However, more research is still needed in this area. From the research carried out, it was possible to know the lean tools most used in the health area, as well as the health care places that have most applied these tools. It would be interesting to carry out new studies that use search strategies that address the relationship of these tools with the different places in which health care is provided. Furthermore, we recommend studies that focus on evaluating the results of long-term interventions. Thus, it would be possible to analyze whether the benefits of these applications persist over time.

It is noticed that the applications of lean healthcare in the health area have evolved so that professionals and researchers use the concept of lean six sigma. The results of this work demonstrated a great use of six sigma tools, such as, for example, DMAIC. The application of this tool has shown several positive results, such as more time dedicated to direct patient care, reduction of unnecessary procedures and lower rate of nosocomial infection, among others. Even so, a greater number of studies on the subject are needed at different levels of health care. It would help to demonstrate its effectiveness in different areas of health, as it was in the industry.

It is reinforced that it is very important that future studies follow a research methodology with a clear study design and sampling in order to increase the level of evidence, strengthening the results of these publications, as well as allowing the reapplication of these methods and tools in other cases.

## Data Availability

Not applicable.

## Appendix A

**Table A1:** Characteristics of publications on the use of Lean tools in order of year of publication.

Year Country Journal	Levels of Evidency	Objectives	Tools	Outcomes
2015 [64] Spain. *Metas Enfermería.,*	Not rated.	Identify the factors that influence waiting times and implement solutions to reduce it.	- Map of steps; - Spaghetti diagram; - Value Stream Map (VSM)	- Increase in added value in the care of pregnant women and women who have recently given birth.
2015 [50] Brazil. *Rev. Lat. Am Enfermagem*	Six.	Compare the application of the Total Quality (TQ) models with another health institution and also with the cases of lean healthcare exposed in the literature.	- 5S; - A3; - Failure Mode and Effect Analysis (FMEA); - VSM; - Plan-Do-Check-Act (PDCA); - *Kaizen.*	- Decreased workload in 70 min; - Increase in the time allocated for assistance of 9 min.
2015 [23] United States. *Prof Case Manag*,	Not rated.	Equip leaders in the field of case management to facilitate alignment with the hospital’s pay-for-performance measures.	- Strengths, Weaknesses, Opportunities, Threats (SWOT).	- Reduction of the patient’s permanence time by 2.6% for managed patients.
2015 [46]. United States. *Cardiovasc Revasc Med*.	Six.	To study the impact of Lean Six Sigma (LSS) in improving the efficiency and transfer of patients in a catheterization laboratory.	- VSM.	- Decrease in length of stay from 20.6% to 17.8%; - Increase in cases with punctual onset from 76.1 to 81.9%; - Increase in cases with ideal shift time from 50.9% to 60.4%.
2015 [44]. Senegal. *Glob Health Action*.	Not rated.	Evaluate how 5S assists in changes in the workplace, in the process and in the results of health services.	- 5S.	- Areas with less unwanted items, better organization and visualization; - More efficient and patient-centered services; - Change in the behavior of the team and patients.
2015 [47]. United States. *Am J Med Qual*, 2015.	Not rated.	Evaluate the implementation of an automated infusion system to reduce waste.	- VSM.	- Reduction in the distance covered by professionals by 14.6%; - Cost increase from US $ 2.68 to US $ 14.61/patient; - Reduction of time to perform tasks in 26 min/day.
2016 [31]. Brazil. *Prod. plan. Control.*	Not rated.	Propose a new approach to value stream mapping that serves as a standard model for Lean applications in hospitals.	- VSM.	- Reduction of treatment time from 187 to 60 days; - Reduction in transitions between departments from 23 to 17; - Reduction, for patients, of trips to the hospital from 9 to 5.
2016 [48]. United States. *Ann Plast Surg,*	Six.	Assess the impact of a Six Sigma program at a medical center.	*-* Voice of the Costumer (VOC); - Suppliers, inputs, process, outputs, customers analysis (SIPOC); - Define, Measure, Analyze, Improve, Control (DMAIC); - Ishikawa Diagram.	- Decreased total operative time from 714 to 607 min; - Decrease in hospital stay by 1.1 days; (6.3 to 5.2 days); - Increase of US $ 1.31 in medical prescription/minute and US $ 3.27 in hospital/minute.
2016 [42]. United States. *J*. *Oncol. Pract*.	Not rated.	Evaluate the chemotherapy prescription process to improve your safety.	- Ishikawa Diagram.	- Increased time spent with the patient by 22 min.
2016 [54]. Saudi Arabia. *Stud. Health Technol. Inform.*	Six.	Improve the use of glucose reagent strips.	- DMAIC.	- Increase in the number of glucometers in the units; - Reduction of unnecessary total executions from 13% to 4%; - Reduction of failed executions from 14% to 7% and reduction of the quality control/patient ratio from 24/76 to 19/81; - Decrease in the total number of strips used in quality control runs by 18.6% and increase in the total number of strips used in patient tests by 10.4%.
2016 [41]. United States. *Mil Med.*	Six.	Improve the mass immunization process.	- VSM; - SIPOC; - 5S.	- Decrease in lead time spent by aspirants on their immunization by 79%; - Reduction of the work team to carry out the immunization process by 10%.
2017 [22]. United States. *Med Care.*	Not rated.	Evaluate the current process and the efficiency standards of the Veterans Choice Program.	- VSM; - 5S; - DMAIC.	- Waste identification; - Recommendations for improvements.
2017 [57] United States. *J Pediatr Surg,*	Not rated.	Determine whether Lean Six Sigma contributes to process improvement when applied simultaneously to all services in an academic hospital.	- SIPOC; - Ishikawa Diagram; - Pareto Diagram; - DMAIC.	- Reduction of the patient’s turnover time from 41 to 32 min; - Decrease in the time between the incision and the application of the surgical dressing from 81.5 to 71 min.
2017 [38] Italy. *J Eval Clin Pract,*	Not rated.	Demonstrate the efficiency of Lean Six Sigma and DMAIC for solutions that allow quality improvement and cost reduction.	- DMAIC; - Gantt Diagram; - Brainstorming; - VSM.	- Reduction of the patient’s residence time by 42%; - Cost reduction of € 260,000/year.
2017 [45] United States. *J Perianesth Nurs.*	Six.	Discuss the implementation of new practices in the preoperative period, along with the benefits and obstacles.	- DMAIC.	- Improved communication between staff, patients and family.
2017 [21] Brazil. *Int J Health Plann Manage.*	Six.	Investigate the history of implementation of Lean Healthcare in Brazil, present particularities of lean implementation in the health sector, especially in developing countries, and suggest relevant topics for future research.	- DMAIC; - VSM; - *Kaizen*; - 5S; - *Kanban*; - *Gemba*; - Spaghetti diagram; - Matrix exchange tool.	- Improved financial performance; - Increased capacity and productivity; - Decrease in lead time.
2017 [51] United States. *J Am Coll Radiol.*	Not rated.	Use the Lean Six Sigma methodology to assess and decrease the waiting time from the outpatient scheduling process to the scheduled procedure.	- VSM; - DMAIC; - FMEA; - Brainstorming; - SIPOC.	- Lead time was not influenced by the day of the week or by the nurse.
2017 [55] Italy. *J Eval Clin Pract*,	Not rated.	Reduce the number of patients affected by bacterial infections.	- DMAIC; - Critical to Quality Tree (CTQ); - SIPOC; - Ishikawa Diagram.	- Reduction in the number of colonized patients from 0.37% to 0.21%; - Decrease in the average number of days of hospitalization.
2017 [49] United States. *Nurs Econ*.	Six.	Examine the impact of Heijunka on the distribution of consultations and decreased waiting times.	- *Heijunka.*	- Increase in the number of patients treated from 3385 to 3738; - Reduction of waiting time; - Increase in revenue.
2017 [39] United States. *Anesth. Analg*.	Not rated.	Resolve deficiencies in the pediatric anesthesia supply chain.	- DMAIC; - VSM; - Spaghetti diagram; - VOC.	- Reduction of the distance covered by the anesthesia technician by 28%.
2018 [52] United States. *Jt Comm J Qual Patient Saf*,	Not rated.	Increase discharge prescriptions to 40% by 10 am and 12% effective patient departures by 12 noon.	- DMAIC; - Flowchart; - Ishikawa Diagram; - Pareto Diagram.	- Increase in discharge prescriptions up to 10:00 from 15.6% to 47.1%; - Increase of the patient’s effective departure until 12:00 from 10.5% to 20.6%.
2018 [30] Brazil. *Einstein.*	Not rated.	Verify the impact of Lean Six Sigma in reducing incorrect entries of inappropriate income and expenses.	- DMAIC; - CTQ; - VOC; - SIPOC; - Ishikawa Diagram; - Pareto Diagram.	- Sigma level increased from 3.44 to 5.92; - Reduction to 0% of inappropriate revenues and expenses, generating savings of R $ 1.8 million.
2018 [53] United States. *Qual. Manag. Health Care*	Not rated.	Reduce expenses by improving the registration process for patient insurance data.	- DMAIC; - SIPOC; - Pareto Diagram; - Brainstorming.	- Reduction in the number of occurrences due to billing errors, non-insurance coverage and incorrect information of 99.6%.
2018 [63] Italy. *J Eval Clin Pract.*	Not rated.	Reduce the risk of healthcare-associated infections through Lean Six Sigma.	- DMAIC; - Ishikawa Diagram.	- Reduction in the percentage of colonized patients from 0.36% to 0.19%.
2018 [24] United States. *Qual Manag Health Care.*	Six.	Identify the root cause of repeated Computed Tomography (CT) losses and implement a solution to increase adherence to the departmental guideline.	- DMAIC; - Ishikawa Diagram. - 5 whys; - FMEA.	- Reduction in the rate of routine repeated CT scans unnecessarily from 15% to 4%.
2018 [29] Brazil. *Leadersh Health Serv*.	Not rated.	Address problems in the flow of patients and identify the causes of waiting time through Lean Healthcare.	- A3; - VSM; - *Gemba*.	- Reduced waiting time by 4.5 h. - Increase by 50% of the complete and correct percentage.
2018 [60] United States. *Int J Health Care Qual Assur*.	Not rated.	Describe the process used to standardize a Program for the Prevention of Violence at Work in a health system.	- Current State Map; - *Poka Yoke*; - VOC; - Just-in-time.	- Increase of requests to appeal the mitigation plan by 500%; - Reduction of the average cycle time from 30 to 10 days; - Increase in the number of trained professionals from 29 to 72.
2019 [20] Brazil. *Qual Manag Health Care*.	Not rated.	Integrate systematic architectural planning techniques with “Lean Healthcare” practices.	- VSM; - Analytic Hierarchy Process (AHP); - First in, first out; - Brainstorming.	- 75% reduction in overtime; - Increased productivity.
2019 [56] Ireland. *Int J Qual Health Care*.	Six.	Optimize nursing time, improve personalized patient care and staff satisfaction.	- SIPOC; - DMAIC; - Spaghetti diagram; - VSM; - 5S.	- Reduction of lead time in 15 min; - Increased patient flow; - Reduction of the admission process to 5 min; - Reduction of patient stay and nursing time.
2019 [25] Ireland. *Clin Radiol*.	Not rated.	To evaluate the use of the Lean Six Sigma methodology to improve the response time for peripherally inserted central catheters.	- VSM.	- Reduction of the time between the request and insertion of the catheter from 3.74 to 1.89 days; - 13.8% increase in radiological activity.
2019 [59] Spain. *Int J Health Plann Manage.*	Six.	Improve work organization, reduce physical effort and obtain greater worker satisfaction.	- VSM; - Spaghetti diagram; - 5S; - *Gemba*; - *Kaizen* week; - PDCA.	- Reduction of the physical space necessary for the performance of work by 44%; - Reduced distance traveled from 318 to 50 m; - Increased professional satisfaction.
2019 [32] United States. *Quality Management in Healthcare*.	Not rated.	Reduce the patient’s cycle time.	- DMAIC; - SIPOC; - FMEA; - Brainstorming.	- Cycle time reduction by 22% (15 min); - Increase in the number of assistance by 27%.
2019 [33] Ireland. *Int J Qual Health Care*.	Six.	Increase the number of diagnoses of Low Grade Mucinous Neoplasms.	- DMAIC; - *Specific, Measurable, Achieveable, Realistic, Time frame* (SMART); - SIPOC; *- Responsible, Accountable, Consulted and Informed* (RACI); - CTQ; - VOC; - Ishikawa diagram; - 5 whys; - FMEA; -*Possible, Implement, Challenge and Kill* (PICK).	- Increase in patients with correct annual access for screening from 61% to 78%; - Increase of patients who received information by 90%, with the creation of the leaflet.
